# Molecular Characterization of Growth Hormone-producing Tumors in the GC Rat Model of Acromegaly

**DOI:** 10.1038/srep16298

**Published:** 2015-11-09

**Authors:** Juan F. Martín-Rodríguez, Jose L. Muñoz-Bravo, Alejandro Ibañez-Costa, Laura Fernandez-Maza, Marcin Balcerzyk, Rocío Leal-Campanario, Raúl M. Luque, Justo P. Castaño, Eva Venegas-Moreno, Alfonso Soto-Moreno, Alfonso Leal-Cerro, David A. Cano

**Affiliations:** 1 Unidad de Gestión Clínica de Endocrinología y Nutrición, Hospital Universitario Virgen del Rocío, Sevilla; 2 Instituto de Biomedicina de Sevilla (IBiS), Hospital Universitario Virgen del Rocío/Consejo Superior de Investigaciones Científicas/Universidad de Sevilla, Sevilla (Spain); 3 Department of Cell Biology, Physiology and Immunology, University of Cordoba, Instituto Maimónides de Investigación Biomédica de Córdoba (IMIBIC), Hospital Universitario Reina Sofia; CIBER Fisiopatología de la Obesidad y Nutrición; and Campus de Excelencia Internacional Agroalimentario (ceiA3), 14014 Córdoba, Spain; 4 Centro Nacional de Aceleradores (Universidad de Sevilla/CSIC/Junta de Andalucía), Sevilla, (Spain); 5 División de Neurociencias. Universidad Pablo de Olavide, Sevilla, (Spain)

## Abstract

Acromegaly is a disorder resulting from excessive production of growth hormone (GH) and consequent increase of insulin-like growth factor 1 (IGF-I), most frequently caused by pituitary adenomas. Elevated GH and IGF-I levels results in wide range of somatic, cardiovascular, endocrine, metabolic, and gastrointestinal morbidities. Subcutaneous implantation of the GH-secreting GC cell line in rats leads to the formation of tumors. GC tumor-bearing rats develop characteristics that resemble human acromegaly including gigantism and visceromegaly. However, GC tumors remain poorly characterized at a molecular level. In the present work, we report a detailed histological and molecular characterization of GC tumors using immunohistochemistry, molecular biology and imaging techniques. GC tumors display histopathological and molecular features of human GH-producing tumors, including hormone production, cell architecture, senescence activation and alterations in cell cycle gene expression. Furthermore, GC tumors cells displayed sensitivity to somatostatin analogues, drugs that are currently used in the treatment of human GH-producing adenomas, thus supporting the GC tumor model as a translational tool to evaluate therapeutic agents. The information obtained would help to maximize the usefulness of the GC rat model for research and preclinical studies in GH-secreting tumors.

Acromegaly is a disorder resulting from excessive production of growth hormone (GH) and consequent increase of insulin-like growth factor 1 (IGF-I), most frequently caused by pituitary adenomas^1^. Elevated GH and IGF-I levels result in wide range of somatic, cardiovascular, endocrine, metabolic, and gastrointestinal morbidities^1^,^2^. If untreated, acromegaly leads to reduced life expectancy due primarily to cardiovascular disease^3^. Achieving biochemical control of the disease restores life expectancy to levels similar to that observed in the general population^4^. Therefore, the main goal of treatment for acromegaly is to normalize both GH and IGF-I levels^5^. Currently available treatment options for acromegaly include surgery, radiotherapy and drug therapy. Three types of medications are available for the treatment of acromegaly: somatostatin analogs, dopamine agonists, and GH receptor antagonists^2^,^6^. However, the currently available therapies fail to control disease activity in a significant number of patients underscoring the need to develop novel therapeutic approaches^7^.

Animal models constitute critical tools for evaluating new therapeutic strategies before clinical testing. Several animal models have been developed to study the effects of chronic GH excess, including exogenous administration of GH, transgenic GH overexpression, and implantation of GH-producing cells^8^,^9^,^10^. The subcutaneous implantation of GH-secreting GC cell line in Wistar Furth rats results in the formation of solid, functional tumors^8^. This acromegaly-like rat model has been successfully used to analyze the effects of chronic GH exposure on target tissues such as cardiac cells, nephrons^11^ and hypothalamic neurons^12^. However, GC tumors remain poorly characterized at a molecular level. In the present work, we report a detailed histological and molecular characterization of GC tumors using immunohistochemistry, molecular biology and imaging techniques that reveal that GC tumors exhibit histopathological and molecular features reminiscent of human GH-producing tumors. We also report proof-of-concept studies with somatostatin analogues that validate the GC tumor model as a translational tool to evaluate therapeutic agents. The information obtained would help to maximize the usefulness of the GC rat model for research and preclinical studies in GH-secreting tumors.

## Results

### Acromegaly features of GC rats are reversible upon surgical removal of tumors

**Figure 1:**
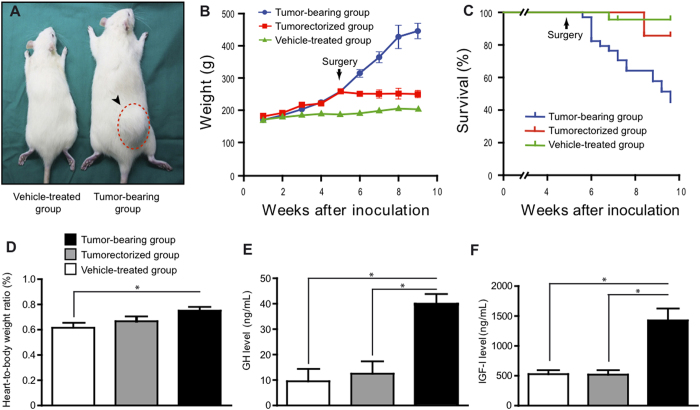
Phenotypic characterization of the acromegaly-like GC rat model. (**A**) Left panel. Representative picture of a rat (right) bearing a GC tumor (circled in red dashed lines) 8 weeks after subcutaneous injection of GC cells compared to a vehicle-treated rat (left). Right panel. (**B**) Increase in body weight after subcutaneous injection of GC cells compared to PBS-injected control rats. Removal of the tumor by surgery blocks the increase in body weight. P < 0.0001. Error bars missing in B is due to the small size of s.e.m. in those data points (**C**) Survival curve of GC tumor-bearing and control rats. GC tumor-bearing rats display increase mortality life expectancy as compared to both tumorectomized and vehicle-treated rats (logrank test’s P < 0.001) (**D**) Increased heart size in GC tumor-bearing rats (9 weeks after GC cells implantation) compared to compared to both tumorectomized (4 weeks after tumor resection) and vehicle-treated rats. Increased blood GH (**E**) and IGF-I levels (**F**) compared to both tumorectomized (4 weeks after tumor resection and vehicle-treated rats. n = 5 samples per group. Data are mean ± s.e.m. *P < 0.05.

Wistar Furth rats implanted with GC cells developed tumors in around 90% of animals injected. GC cells-grafted rats show a remarkable increase in body weight two weeks after cell implantation, as compared to vehicle-treated rats (Fig. 1A,B). Body weight significantly decreased after tumor removal, reaching equivalent body weight to age-matched vehicle-treated rats. Naive tumor-bearing rats showed reduced life expectancy (median life expectancy = 9 weeks after GC cell implantation) as compared to both tumorectomized and vehicle-treated rats while survival curves of tumorectomized rats did not differ from vehicle-treated rats (Fig. 1C). As previously documented^8^,^12^, increased size was observed in a number of organs, namely spleen, and heart in GC tumor-bearing rats. After tumor resection, the size of these organs reverted to normal levels (Fig. 1D and Supplementary Table 1). Naive tumor-bearing rats showed elevated serum levels of GH and IGF-I, while normal levels of these hormones were found in both tumorectomized and vehicle-treated rats (Fig. 1E,F). Normal serum prolactin levels were found in tumor-bearing rats confirming that GC tumors produce exclusively GH (Supplementary Figure 1).

### Tumor growth kinetics of GC tumors

**Figure 2:**
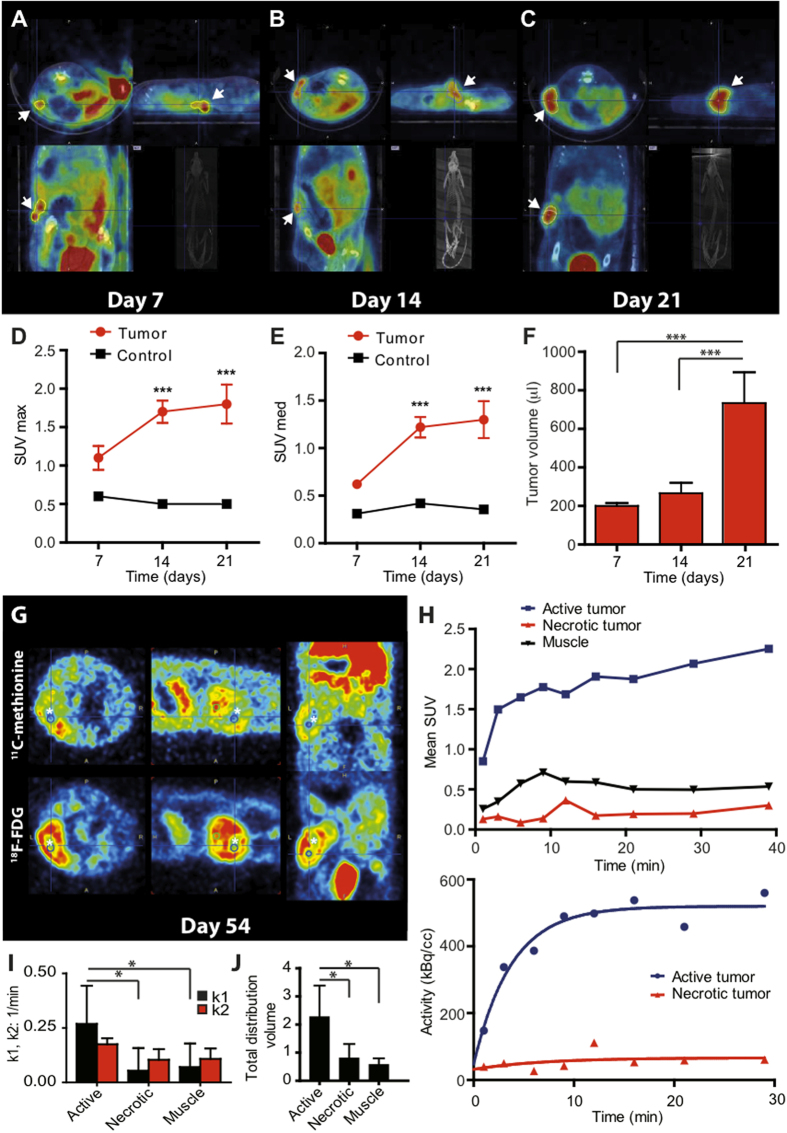
Molecular imaging with FDG- and Met-PET/CT. Representative [^18^F]FDG images (transverse, sagittal, lateral) of established GC tumors after 7 (**A**), 14 (**B**) and 21 (**C**) days after subcutaneous injection of the cells. A longitudinal CT section is also shown in right bottom panels. White arrowheads mark the localization of the tumor. [^18^F]FDG uptake expressed as maximum standard uptake value (SUV) (**D**) and mean value (**E**) of established GC tumors after 7, 14 and 21 days after subcutaneous injection of the cells. (**F**) Tumor volumes derived from SUV data in GC tumor. (**G**) Representative [^11^C]Met-PET (top) and [^18^F]FDG -PET (bottom) images (transverse, sagittal, lateral) of GC tumors 54 days after subcutaneous injection of the cells. Asterisks mark areas with reduced metabolic activity. (**H**) Top graph- Time activity curves of [^11^C]Met-PET/CT: red- necrotic zone of the tumor, blue-active area of the tumor, black—muscle in contralateral hind leg. Bottom graph. Time-activity curve of the active and necrotic zone of the tumor showing tracer accumulation over the first approximately 10 min, followed by a plateau phase (plateau value: 559.7 kBq/cc; 95% confidence interval [479.4–640]). The data of the active area of the tumor fitted to a one phase association model (R^2^: 0.88; P < 0.05). (**I**) Kinetic analysis performed by fitting a standard 2-parameter, one-tissue compartment model to the dynamic PET data. k1 (transport from arterial plasma to tissue); k2 (transport from tissue to arterial plasma). K1 values are higher in the active part of the tumor active part and the necrotic and between active part and the muscle. (**J**) Same data for Vt, total distribution volume. *P < 0.05.

Longitudinal noninvasive imaging allows monitoring of tumor growth providing a useful tool for evaluation of therapeutic agents. To visualize early tumor development and monitor tumor growth in GC rats, we used [^18^F]FDG-PET and [^11^C]Met-PET, combined with computerized tomography. An increase in maximal and median standard uptake value (SUV med) at the site of the injection in GC rats compared to vehicle-treated rats was observed as early as 7 days after cell implantation although this increase was not statistically significant (Fig. 2A,D,E). One week later, when tumors became palpable, FDG uptake at the site of the GC cell injection was significantly increased (Fig. 2B,D,E). This increase in FDG uptake in GC rats persisted three weeks after the cell injection (Fig. 2C,D,E). At this time point, a significant increase in tumor volume was found (Fig. 2F). GC tumors continued to grow over several weeks and developed areas with low FDG uptake (Fig. 2G, bottom). To monitor metabolic activity in these large tumors, [^11^C]Met-PET, which provides more sensitivity in pituitary adenomas that are characterized by elevated amino acid metabolism, was performed. Eight weeks after the injection, tumor metabolic activity was predominantly located at the periphery of the tumors (Fig. 2G top), in agreement with the central necrotic areas observed at gross examination (see below). Using a 1-compartment model to fit PET data, two different regions with distinctive [^11^C]Met uptake were identified in the tumors. Areas with high metabolic activity were mainly localized at the periphery of the tumor while areas with reduced metabolic activity were found in the center of the tumor (Fig. 2G top, H). Thus, the pattern of high protein synthesis determined by [^11^C]Met-PET/CT (Fig. 2G top) is similar to the pattern of glucose metabolism determined by [^18^F]FDG-PET/CT (Fig. 2G bottom). The kinetic modeling of [^11^C]Met uptake using one tissue compartment model showed that the unidirectional transport of the tracer from blood compartment to the first tissue compartment K1 (Fig. 2I) as well as total distribution volume Vt (Fig. 2J) was higher in active tumor tissue compared to necrotic tumor tissue or muscle control tissue. Thus, kinetic modeling of PET data in GC rats indicates that GC tumors develop large areas with low metabolic activity after several weeks of growth.

### Histological characterization of GC tumors

**Figure 3:**
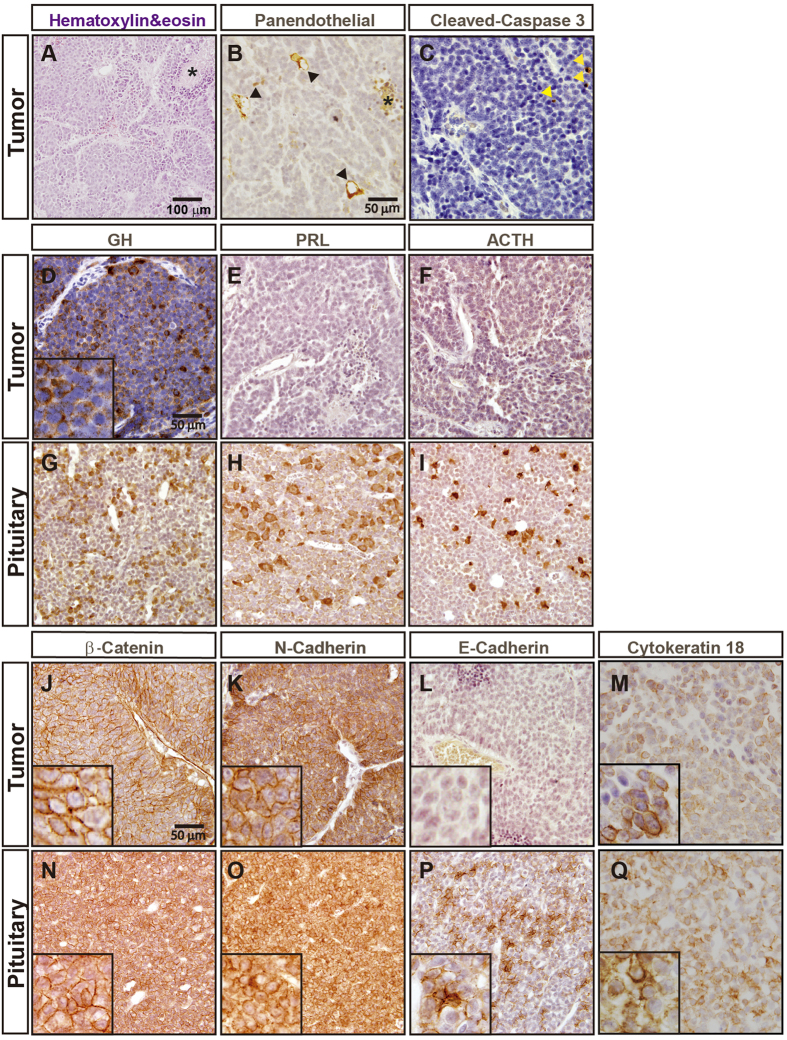
Histological characterization of GC tumors. (**A**) Hematoxylin/Eosin staining of 3-week-old GC tumor sections shows glandular regions composed of cells displaying typical endocrine cell morphology (inset) arranged in clusters. Areas of necrosis are observed (asterisks). (**B**) GC tumor cells arrange in clusters around blood vessels as shown by immunohistochemical staining for PECAM-1. Asterisks indicate areas of necrosis. (**C**) A few, scattered, apoptotic cells (arrowheads) are observed in GC tumors. GC tumors are composed exclusively of GH-producing cells (**D**). No prolactin (**E**) and ACTH (**F**) accumulation is observed in GC tumors. Robust expression of GH (**G**), prolactin (**H**) and ACTH (**I**) is found in normal rat pituitary. (**J–O**) Accumulation of cell adhesion proteins in GC tumors. Robust ß-catenin (**J**), N-cadherin (**K**) accumulation is observed in GC tumor cells. Almost no accumulation of E-Cadherin is observed in GC tumors cells (**L**). Robust accumulation of ß-catenin (**M**), N-cadherin (**N**) and E-cadherin (**O**) in normal rat pituitary. Perinuclear accumulation of keratin 18 in GC tumors (**M**) and normal rat pituitary (**N**).

Gross morphology analysis of GC tumors revealed well-defined, solid, encapsulated tumors (Supplementary Figure 2). No distant metastases were observed in GC tumor-bearing rats at any time point analyzed (out of more than 30 rats analyzed). Histological analysis of 3-week-old tumors revealed glandular regions composed of cells displaying typical endocrine cell morphology (small round cells with a big nucleus, highly acidophilic) (Fig. 3A) arranged in clusters around big blood vessels (Fig. 3A,B). Consistent with the relatively rapid growth observed with the imaging data, GC tumor cells were highly proliferative (Ki-67 index: 31.7 ± 8.8) (Supplementary Figure 3). However, small areas of necrotic tissue were found adjacent to glandular tissue (Fig. 3A,B). Tumors at later stages display a marked increase in the size and number of necrotic areas (Supplementary Figure 4). This increased cell death observed in GC tumors did not appear to be due to massive cell apoptosis; only a few, scattered apoptotic cells were found in tumors sections (Fig. 3C). As expected, GC tumors were composed exclusively of GH-producing cells; no immunoreactivity for other pituitary hormones such as prolactin and ACTH was detected (Fig. 3D–I). To provide a further histological characterization of GC tumors, we analyzed the expression of cell adhesion proteins which have been associated with major features of pituitary adenomas such as size, proliferation and invasion^13^. The expression pattern of the cell adhesion molecules ß-catenin and N-cadherin in GC tumor cells were similar to that observed in normal rat pituitary (Fig. 3J,K,N,O). However, no expression of E-cadherin was observed in GC tumor cells (Fig. 3L,P). The sparsely granulated subtype of human GH-producing pituitary adenomas display decreased E-cadherin expression. Based on GH immunoreactivity, GC tumors seem to be more similar to the human densely granulated subtype of human GH-producing pituitary tumors. Densely exhibit strong, diffuse cytoplasmic accumulation of GH compared to the focal and weak GH immunoreactivity typical of sparsely granulated somatotroph adenomas^14^. Sparsely granulated somatotroph adenoma type can be unequivocally identified by the presence of fibrous bodies (also called globules) composed of keratins 8/18^14^. Immunohistochemistry for keratin 18 in GC tumors did not reveal the presence of fibrous bodies. keratin 18 exhibited perinuclear accumulation similar to that found in normal rat pituitary (Fig. 3M,Q).

### Senescence and cell cycle regulation analysis of GC tumors

**Figure 4:**
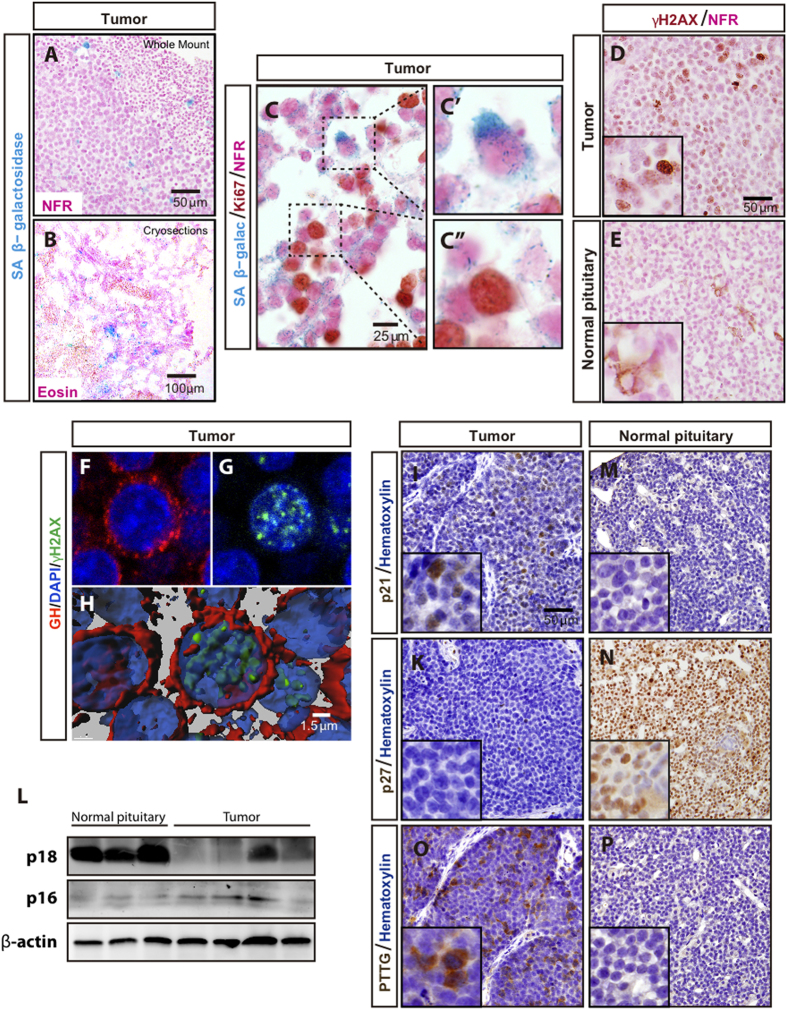
Senescence and cell cycle proteins in GC tumors. Robust Senescence-associated β-galactosidase (SA-β-Gal) accumulation was observed in GC tumors both in paraffin sections of whole mount SA-β-Gal-stained tumors (**A**) and in cryogenic sections stained for SA-β-Gal (**B**). SA-β-Gal accumulation was observed only in non-proliferative GC tumor cells (**C**). The boxed areas are shown in higher magnification in C’ and C”. Increased, nuclear accumulation of the DNA damage response protein γH2AX in GC tumors (**D**) compared to normal rat pituitary (**E**). Note that a few pituitary cells display cytoplasmic (likely unspecific) γH2AX signal. Confocal microscope analysis showing GH/γH2AX co-localization in GC tumor cells (**F–H**). (**H**) 3D reconstruction of the Z-stack for the pictures shown in (**F,G**). Increased accumulation of cyclin-dependent kinase inhibitor p21 in GC tumors (**I**) compared to normal rat pituitary (**M**). No expression of cyclin-dependent kinase inhibitor p27 is observed in GC tumors (**K**) but it is homogeneously expressed in normal rat pituitary (**N**). (**L**) Western blot analysis of p16 and p18 in 3 independent normal rat pituitary samples and 4 independent GC tumor samples. PTTG1 is overexpressed in GC tumors (**O**) compared to normal rat pituitary (**P**).

Increased senescence has been hypothesized to explain the benign nature of human pituitary adenomas^15^,^16^. To determine whether cellular senescence was activated in GC tumors, we performed senescence-associated β-galactosidase (SA-β-Gal) staining on whole-mount as well as frozen sections of GC tumors. A prominent SA-β-Gal staining was observed in GC tumors (Fig. 4A,B). As expected, SA-β-Gal activity was observed only in non-proliferative tumoral GC cells (Fig. 4C). Robust accumulation of another surrogate marker of senescence, the DNA damage response protein γH2AX, was found in GC tumors (Fig. 4D,E). Co-localization of γH2AX and GH was determined by thorough confocal microscope analysis confirming that GC tumor cells display activation of senescence markers (Fig. 4F–H and Supplementary Figure 5). Cellular senescence is associated with the activation of cell cycle inhibitors^16^. In agreement with this, we observed robust expression of the cyclin-dependent kinase inhibitor p21 in GC tumors whereas no expression was detected in normal rat pituitary (Fig. 4J,M). In contrast, the expression of the cyclin-dependent kinase inhibitor p27 was markedly reduced in GC tumors compared to normal pituitary (Fig. 4K,N). Germ-line mutations in Cdkn1b gene encoding the p27 protein predispose to pituitary adenoma formation in both rats and humans^17^. Sequencing of the Cdkn1b gene in GC tumors, performed as previously described^17^, did not reveal any mutations. Two other cyclin-dependent kinase inhibitor, p16 and p18, has also been reported downregulated on pituitary adenomas^18^. Western blot analysis also revealed a downregulation of p18 expression in GC tumors compared to normal rat pituitary (Fig. 4L and Supplementary Figure 6). p16 levels were similar between GC tumors and pituitary (Fig. 4L and Supplementary Figure 6). Overexpression of pituitary tumor-transforming gene 1 (PTTG1) is frequently observed in human GH-secreting tumors and it has been suggested to be related to induction of cellular senescence^19^. In concordance with these human studies, GC tumors exhibit increased PTTG1 expression compared to normal rat pituitary (Fig. 4O,P and Supplementary Figure 7).

### Effect of somatostatin inhibitors on primary cell cultures from GC tumors

**Figure 5:**
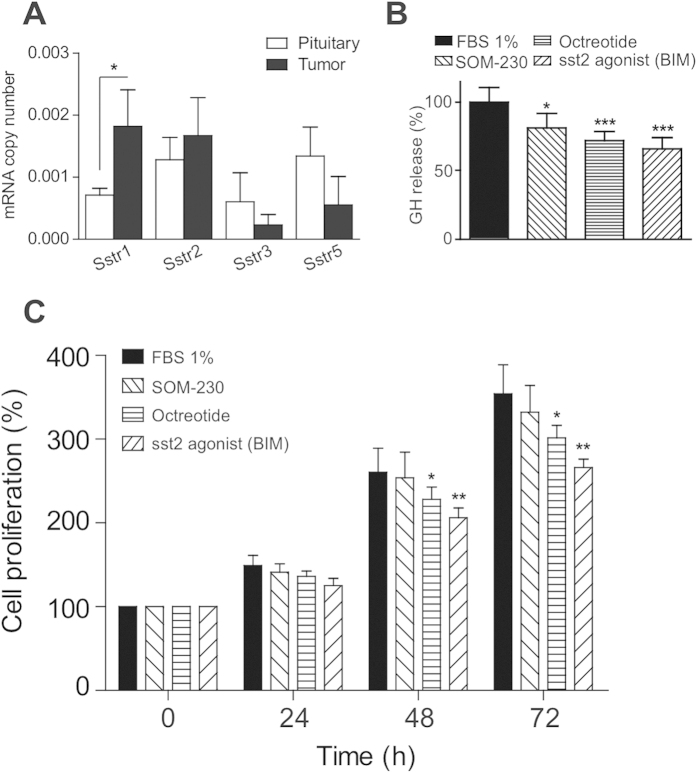
Expression profile and effect of somatostatin analogues on GH secretion and cell proliferation in cultures from GC tumors. (**A**) Expression profile of somatostatin receptor (Sstr) subtypes in GC tumors (n = 5). mRNA copy number was adjusted by expression of a control gene (beta-actin) (**B**) Effect of 4 h treatment with somatostatin analogues (SOM-230, Octreotide and BIM-23120) at 100 nM on GH secretion in cultures from GC tumors. Values are expressed as percent of fetal bovine serum (FBS)-treated control cells (set at 100%; n = 3; 3–5 wells/treatment). (**C**) Cell viability in culture of GC tumors treated with somatostatin analogues (SOM-230, Octreotide and BIM-23120) at 100 nM for 24, 48 and 72 hours as compared with FBS-treated control cells. Values are expressed as percent of FBS-treated controls (set at 100%; n = 3; 3–5 wells/treatment). Data are mean ± s.e.m. *P < 0.05, **P < 0.01, ***P < 0.001.

To evaluate the usefulness of GC tumors as a model for testing of therapeutic agents, we decided to determine whether GC tumor cells were sensitive to somatostatin analogues. Somatostatin analogues are the primary current medical treatment for GH-producing pituitary adenomas acting primarily as inhibitors of GH secretion (although tumor growth might also be affected)^6^,^7^. As a first approach, we examined somatostatin receptor expression in GC tumors by quantitative real time PCR analysis. Sstr1 and Sstr2 were highly expressed followed by Sstr5 and Sstr3 (Fig. 5A). As a comparison, we measured somatostatin receptor expression in normal rat pituitary. Sstr5 and Sstr2 displayed the highest expression followed in normal rat pituitaries followed by Sstr1, and Sstr3. Sstr1 was the only Sstr subtype that displayed a statistically significant difference in gene expression between GC tumors and normal rat pituitary (Fig. 5A). We also measured the expression of the receptor for the Growth hormone-releasing hormone (GHRH), another hormone-regulatory pathway suggested to be implicated in GH-secreting adenoma formation^20^. Although we detected expression of GHRH receptor in normal pituitary, almost no expression could be observed in GC tumors (Supplementary Figure 8). We next determined the *in vitro* response of GC tumor cells to somatostatin analogues. To this end, primary cell cultures were obtained from 3-week-old GC tumors and the effect of the somatostatin analogues SOM-230 (multireceptor ligand), octreotide (higher affinity to Sstr2) and BIM-23120 (Sstr2 specific) on GH secretion and cell proliferation was measured. The three somatostatin analogues significantly inhibited GH release in GC tumor cultures (Fig. 5B), with BIM-23120 showing the highest inhibitory effect (34.3%) followed by octreotide (28.3%) and SOM-230 (19%). A statistically significant decrease in cell proliferation was observed in GC tumor cultures treated with octreotide and BIM-23120, but not with SOM-230 (Fig. 5C). Altogether these results indicate that GC tumors are sensitive to somatostatin analogues.

## Discussion

Preclinical animal models play a central role in understanding the biology and pathophysiology of tumor formation as well as in the testing of therapies. There is a paucity of animal models that faithfully recapitulate GH-secreting tumors^21^. The GC acromegaly-like rat model remains largely under-recognized in the field of pituitary tumor research. We have revisited the GC rat model to perform an extensive histopathological and molecular characterization of the GC tumors that should further facilitate the use of this model by the pituitary research community.

The phenotypic data of the GC rats reported here are in agreement with previous findings^8^,^12^ and further clarify the phenotype of this acromegaly animal model. GC cells-grafted rats display a rapid increase in body length and weight, as early as two weeks after cell implantation. This increase in body weight is concomitant with a marked elevation in serum levels of GH and IGF-I. In addition to increased body weight, GC cells-grafted rats display other classical human acromegaly traits such as splenomegaly and enlargement of the heart. Increased mortality was observed in GC tumor-bearing rats. Autopsy analysis could not conclusively demonstrate the underlying cause of this increased mortality but cardiovascular and respiratory disease might be associated^8^, as it has been previously shown in human acromegaly and transgenic mouse models of acromegaly^3^,^10^,^22^. Nevertheless, the increased mortality of GC rats seems to be directly due to the presence of the tumor since tumorectomized rats do not display reduced survival. The resection of the tumor causes a relatively drop of body weight in GC rats, reaching levels similar to those observed in control rats within a few weeks. This decrease in body weight was concomitant with a marked reduction of serum GH and IGF-I levels. These results are in agreement with studies showing that the body weight of patients with GH-producing adenomas rapidly decrease after successful surgical treatment^23^. Tumor resection also causes normalization in the weight of organs of GC tumor-bearing rats including the spleen and heart. No analysis of cardiac function in GC rats was performed in our study but it is tempting to speculate this normalization in the weight of the heart might be associated with improvement in cardiac function as it has been shown in human patients after successful biochemical control of acromegaly^24^. Nevertheless, this phenotypic analysis illustrate how the GC rat model, in contrast to other animal models of GH excess such as GH-overexpressing transgenic mice, offers the advantage of allowing to study the sequelae caused by excess of GH/IGF-I levels even after normalization of the circulating GH/IGF-I levels by surgical removal of the tumor.

In our hands, Wistar Furth rats implanted with GC cells developed palpable tumors between 2–3 weeks after cell implantation. In agreement with this, significant increases in [^18^F]FDG consumption in GC tumors were observed starting from the second week after cell implantation. There was a trend towards increased [^18^F]FDG signal as early as 7 days after cell implantation but it was not statistically significant, probably due to the relatively small number of rats analyzed. [^18^F]FDG-PET imaging also allowed us to non-invasively measure the volume of the GC tumors finding a marked increase in tumor volume between 2 and 3 weeks after cell implantation. GC tumors continued to grow over several weeks. In these large tumor xenografts, regions of higher and lower [^18^F]FDG consumption were observed. Kinetic modeling of [^11^C]Met uptake confirmed the formation of areas with low metabolic activity on 6–8 week-old GC tumors. This decrease in metabolic activity is likely due to the formation of internal tumor necrosis within these relatively large xenografts. Thus, based on our micro PET-CT results, three stages of tumor progression in the GC rat model could be broadly defined. During the first two weeks after GC cell implantation increased metabolic activity is observed in tumoral tissue. A marked increased in tumor volume occur after three weeks of growth. At later stages (6–7 weeks after cell implantation), internal areas with decreased metabolic activity (likely necrotic areas as evidenced by histological observations) are developed in the tumors. Thus, this pattern of the progression of GC tumor growth should be taken into account when evaluating therapeutic agents in this rat model of acromegaly. PET scans have shown to be a valuable technique for the diagnosis and management of pituitary adenomas^25^,^26^. Our PET results obtained in GC tumors are consistent with studies performed in human subjects that demonstrate that GH-secreting adenomas display high metabolic activity assessed by both [^18^F]FDG-PET and [^11^C]Met-PET imaging^25^,^26^,^27^,^28^.

Histological examination demonstrated that subcutaneously grown GC tumors recapitulate the morphology of human pituitary adenomas. Typically, GC tumor cells were relatively small with a high nuclear/cytoplasmic ratio. Based on GH immunoreactivity and keratin 18 accumulation, GC tumors are reminiscent of the human densely granulated subtype of human GH-producing pituitary tumors. In humans, pituitary adenomas that produce both GH and prolactin, and (more rarely) both GH and ACTH, are found^20^,^29^. GC tumors were composed exclusively of GH-producing cells with no evidence of activation of expression of prolactin or ACTH at any of the stages analyzed, consistent with what has been previously reported for GC cells grown under standard cell culture conditions^30^. Thus, the GC rat model represents a homogeneous *in vivo* model of GH-producing adenomas. GC tumor cells appear to arrange in clusters around blood vessels. Regions of the tumors distant from blood vessels contain necrotic areas. These necrotic areas are particularly prominent in advanced stages when the size of the tumors is markedly increased consistent with the micro PET-CT findings showing areas with lower metabolic activity. These results suggest that lack of blood flow may inhibit GC tumor growth and lead to necrosis, likely due to hypoxia and/or lack of nutrients.

Altered expression of cell adhesion proteins such as N-cadherin, E-cadherin and ß-catenin has been associated with the formation of pituitary adenomas^13^. Our immunohistochemical analysis of these proteins in GC tumor cells revealed strong, membranous expression of N-cadherin and ß-catenin, similar to that observed in normal rat pituitary gland (our data and^31^). However, expression of E-cadherin appears greatly reduced in GC tumors. Interestingly, decreased E-cadherin expression has been reported in different types of human pituitary adenomas, including GH-producing adenomas^32^,^33^. This low expression of E-cadherin appears to be linked to increased invasiveness in human somatotroph pituitary adenomas^32^. Despite the low expression of E-cadherin displayed by GC tumors, no signs of malignant transformation or local invasiveness are observed in GC cells-grafted rats. Thus, GC tumors display a benign behavior similarly to what it is commonly observed in human somatotroph pituitary adenomas (or other types of pituitary adenomas, for that matter)^34^. It has been suggested that activation of cellular senescence might underlie the generally benign nature of pituitary adenomas compared to other types of cancer^15^,^16^. Thus, senescence markers such as cyclin-dependent kinase inhibitor p21 and senescence-associated β-galactosidase are induced in most of human GH-secreting adenomas, but not in aggressive pituitary carcinomas^35^,^36^. In agreement with this notion, GC tumors display induction of p21 expression and senescence-associated ß-galactosidase activity. In the majority of human GH-producing pituitary adenomas, p21-dependent senescence is associated with *Pttg-1* overexpression^36^. In agreement with this human data, GC tumors display robust PTTG-1 expression. In contrast to the robust expression of the cyclin-dependent kinase inhibitor p21, a dramatic reduction in the expression of the cyclin-dependent kinase inhibitor p27 is observed in GC tumors. Although p27 expression is down regulated in pituitary adenomas^37^,^38^ it should be noted that the majority of GH-secreting adenomas display p27 accumulation. Downregulation of two other cyclin-dependent kinase inhibitor, p16 and p18, has also been reported on pituitary adenomas^18^. Western blot analysis revealed a downregulation of p18 expression in GC tumors compared to normal rat pituitary. p16 levels were similar between GC tumors and pituitary but we should note the p16 levels were very low in both cases and thus, we have might not been able to detect subtle differences. Thus, our data indicate that GC tumors display alterations in the expression of several cell cycle related genes. Some of these cell cycle proteins display a pattern reminiscent of that observed in human GH-secreting adenomas while others not. Altogether, our results are consistent with the strong body of evidence that indicates that cell cycle deregulation is significantly involved in pituitary tumorigenesis (for reviews, see^18^).

Inhibition of somatostatin receptor (SSTR) activity by somatostatin analogues is the main medical treatment for GH-producing pituitary adenomas^2^,^6^,^7^. The expression pattern of SSTR subtypes appears to correlate with the response to treatment with somatostatin analogues^39^,^40^,^41^. Indeed, quantification of SSTR subtype expression is currently being explored as a potential prognostic marker in human GH-secreting adenomas^42^. In GH-secreting adenomas, SSTR2 and SSTR5 are predominantly expressed followed by SSTR3 and SSTR1, while SSTR4 is virtually undetectable^40^,^43^,^44^. Rat GC tumors display a somewhat similar expression pattern of Sstr subtypes, but Sstr1 expression in GC tumors was higher compared to what it has been observed in human GH-secreting adenomas. The elevated Sstr1 expression in GC tumors does not seem to be due to species differences since the Sstr expression pattern in normal pituitary is similar to that described in normal human pituitary^45^. Indeed, Sstr1 was the only Sstr subtype that displayed a statistically significant difference in expression between GC tumors and normal rat pituitary. The relevance of this high Sstr1 expression in GC cells on GH secretion is unclear since conflicting results have been reported. A previous study performed in GC cells in culture reported that inhibition of Sstr1 efficiently blocked GH release^46^ but no Sstr1 binding was detected in radioligand binding experiments^47^. It is interesting to note that despite the general low SSTR1 expression levels observed in GH-secreting adenomas, SSTR1 selective agonists have been shown to inhibit GH release from human GH-secreting adenomas *in vitro*^48^,^49^. Thus, these functional studies suggest that SSTR1 may also play a role in the regulation of GH secretion in human GH-secreting adenomas. Nevertheless, our Sstr expression results in GC tumors obtained by quantitative RT-PCR are in agreement with the Sstr expression pattern observed in GC cells grown under standard culture conditions by other techniques such as fluorescent immunolabelling assays^50^. Increased expression of the GHRH receptor, (another hormone-regulatory pathway that might be involved in GH-secreting adenoma formation^20^ has been reported in agressive somatotroph adenomas^51^. We could not detect Ghrh expression in GC tumors indicating that this pathway does not play a significant role in GC tumor growth. To this regard, it is important to mention that a truncated form of the GHRH with defective function have been identified in GH-producing pituitary adenomas^52^, thus raising questions about the relevance of this pathway in pituitary adenomas.

To further validate the GC model as a translational tool to evaluate therapeutic agents, we established primary cell cultures from GC rat tumors and tested the effect of three different somatostatin analogues in GH secretion and cell proliferation. The somatostatin analogues chosen display slightly different affinity patterns to somatostatin receptor subtypes. Octreotide is a clinically used somatostatin analogue that binds primarily to SSTR2 and to a lesser extent to SSTR5, BIM-23120 exhibits a very specific affinity to SSTR2, whereas SOM-230 is a multi-somatostatin receptor ligand with high affinity for SSTR1, SSTR2, SSTR3 and SSTR5^42^,^53^,^54^. These somatostatin analogues have been previously shown to inhibit GH secretion in primary cultures of human GH-secreting adenomas (for reviews, see^55^,^56^). In keeping with these observations, all somatostatin analogues cause a marked decrease in GH secretion in GC tumor cells. BIM-23120 and octreotide exhibit the highest inhibitory effect on GH secretion in GC tumors cells, likely due to the elevated expression of Sstr2 observed in GC tumors. Besides the inhibitor effects on GH secretion, somatostatin analogues display an antiproliferative effect in primary cultures from human GH-producing pituitary adenomas (for reviews, see^57^). In GC primary tumor cultures we observe that octreotide and BIM-23120, but not SOM-230, cause a decrease in cell proliferation. Interestingly, although SOM-230 exhibits an inhibitory effect on GH secretion, yet it does not affect cell proliferation in GC primary cultures. To this regard, it has been previously shown that the inhibition of cell proliferation by somatostatin analogues might occur independently of the effects on GH secretion in dispersed human GH-secreting pituitary adenoma cells *in vitro*. This dissociation of effects on GH secretion and cell proliferation by somatostatin analogues might be due to differences in receptor concentration and the SSTR subtypes expressed^58^. Although *in vivo* studies with somatostatin analogues such as BIM-23120 or SOM-230 are needed to fully validate the GC rat model for pharmacological studies, our *in vitro* experiments constitute simple proof-of-concept studies that support the use of the GC bearing-tumor rats for the testing of therapeutic agents.

In summary, despite the obvious experimental differences between human GH-secreting adenomas and the GC tumors, we believe that our observations warrant the use of the GC tumor bearing rats as an animal model to study GH-secreting adenomas, the deleterious effects caused by prolonged exposure to elevated circulating GH/IGF-I levels as well as the phenotypic changes that occur after normalization of the circulating GH/IGF-I levels by surgical or phamarcological treatment.

## Material and Methods

### Animal care, graft induction and surgery

All procedures involving experimental animals were performed in accordance with local animal welfare laws, guidelines and policies. All experimental protocols were approved by the IBiS-Virgen del Rocio Ethics Committee. Female Wistar Furth rats were purchased from a commercial supplier (Charles River Laboratories, L’Arbresle, France). GC cells were obtained from Jacques Epelbaum (INSERM, Centre de Psychiatrie et Neurosciences, Université Paris Descartes) and culture conditions have been described elsewhere^8^. GC tumors were generated were by subcutaneous injection of 1 × 10^7^ GC cells (in 0.3 ml of PBS) in the flank of 9-weeks-old rats. Control (vehicle-treated) rats were injected with PBS. For surgical removal of the tumor, rats were anesthetized with ketamine hydrochloride (40 mg/kg; ip) plus xylazine (8 mg/kg; im). Sham surgery involved an incision on the contralateral side of the tumor. Follow-up study endpoint was defined as the time that any group (tumorectomy, naive tumor, or saline) had reached 50% of survival. Pituitaries and tumor samples were obtained from these animals for histology, immunohistochemistry and quantitative real time PCR analysis.

### [^18^F] FDG and [^11^C]MET PET/CT

Commercial IBA-Molecular Flucis® [^18^F]fluorodeoxyglucose [^18^F]FDG was used for glucose metabolism evaluation. The radioligand [^11^C]L-Methionine [^11^C]Met was prepared by reaction of L-homocysteine thiolactone hydrocloride with [^11^C] Methyl iodide as previously reported^59^ but [^11^C] Methyl iodide was obtained in gas form. The decay-corrected radiochemical yield was about 32% (End of Bombardment), the radioactive concentration was 3.97 ± 0.19 GBq/mL at the moment of injection, and radiochemical purity was greater than 99%. pH was at pharmacopoeia range (4.5–8) in all batches. A dose of 50–100 MBq for each rat was used for all radiotracers. PET scans were performed with a high-resolution Philips Mosaic HP preclinical PET system (Philips Medical Systems, Eindhoven, The Netherlands) with a field of view of 128 × 120 mm. Before tracer injection, rats were pre-warmed to a body temperature of 37 °C and anaesthetised with inhalation of isoflurane-oxygen gas mixture (2% isoflurane, 0.5 l/min oxygen). Rats were kept under anaesthesia for the entire experimental procedure using a Minerve Animal Anaesthesia System (Equipement Vétérinaire Minerve, Esternay, France). Body temperature was maintained at 37 °C throughout the uptake period. Radiotracers were administered intravenously through a custom-made catheter placed in the lateral tail vein. Static PET imaging was performed 45 min after [^18^F]FDG injections. Three full body scans (3 × 15 min) starting from tumor location in the hind leg was performed. For dynamic [^11^C]Met-PET imaging, rats were injected at the start of the acquisition. CT scans were performed using a Bioscan NanoCT scanner (Bioscan, Paris, France) after each PET scan using a transferable animal bed so that animals were kept in the same position to allow accurate subsequent fusion of PET and CT image datasets. Images were acquired at 45 kVp, 123 μA current with 500 ms exposition, 240 projections per rotation that permitted uniform 0.2 mm image resolution. PET and CT images were fused within PMOD program (version 3.309). CT images were calculated in Houndsfield units, and PET images in SUV units. An ellipsoidal VOI was drawn around the tumor, moved and rotated to encompass full tumor and to avoid nearby regions with high activity. Automatic VOI was calculated at the level of 50% of maximum standardized uptake value (SUV) minus minimum SUV in the elliptical initial region. The calculated VOI was used as an estimation of tumor volume, x-y-z dimensions and maximum FDG. Quantitative PET imaging results are also reported in terms of with SUV median (median of pixel SUV values within a defined region of interest). For kinetic analysis with [^11^C]Met, distinct regions of interest were drawn from the center of the tumor, the periphery and the muscle of the left hind leg. Time–activity curves were calculated for each of these regions and expressed as a mean SUV and KBq/cc. Kinetic analysis was performed by fitting a standard 2-parameter, one-tissue compartment model to the dynamic PET data as previously described^60^. The parameters calculated included transport from arterial plasma to tissue (k1) and transport from tissue to arterial plasma (k2). V_T_ was calculated as k1/k2. The whole blood image-derived input function used for kinetic calculation was obtained from the tail vein of the rat.

### Hormone measurements

For *in vivo* hormone measurements, blood from the subclavian vein was collected and centrifuged to obtain serum. GH, IGF-I and prolactin levels were measured by ELISA commercial kits (EZRMGH-45K, Merck Millipore, Madrid, Spain; AC-18F1, Immunodiagnostic Systems, Ortho Clinical Diagnostics, Rochester, USA; A05101, Bertin Pharma, Montigny le Bretonneux, France).

### Tissue preparation, Histology, Immunohistochemistry and Microscope Analysis

Tumor specimens were fixed in 4% (wt/vol) paraformaldehyde (PFA) in phosphate-buffered saline (PBS) at 4 °C overnight. Pituitaries were fixed in 4% PFA in PBS at room temperature for 1 hour. Tissues were washed three times in PBS, rinsed in 70% ethanol and processed for paraffin embedding in a tissue processor (ASP200S, Leica, Barcelona, Spain). Immunohistochemical analysis was performed as previously described^61^. Briefly, following dewaxing, rehydrating and pressure-cooker antigen retrieval, paraffin sections were permeabilized in 0.2% Triton X-100 in PBS (PBT) and blocked in 3% donkey serum in PBT (60 minutes at room temperature). Tumor and pituitary sections were then incubated with primary antibodies at 4 °C overnight, washed three times in PBS and incubated with secondary antibodies coupled with the appropriate conjugated secondary IgG antibody (45 minutes at room temperature). Primary antibodies are listed in supplementary material Table S2. Staining for diaminobenzidine (DAB) was performed with the ABC Elite immunoperoxidase system (Vector Laboratories). When further amplification was required, the EnVision enzyme-conjugated polymer (DAKO, Barcelona, Spain) was used. Sections were counterstained with Mayer’s hematoxylin (Bio Optica, Milan, Italy), dehydrated and mounted with DPX mountant for histology (Sigma-Aldrich, Madrid, Spain). Hematoxylin/eosin staining was performed as previously described^62^. Bright field images were acquired using a BX61 microscope (Olympus, Japan). All photomicrographs shown are representative of at least three independent samples. Confocal images were acquired with a LSM-7 DUO microscope (Zeiss, Germany). For 3D reconstruction, Z-stack confocal images were reconstructed using Imaris (Bitplane).

### SA-β-galactosidase Activity

SA-β-Gal staining was performed in whole-mount tumors or in tumor cryosections preserved in OCT. For whole-mount staining, freshly dissected tumors were cut into 1–3 mm pieces and fixed with a solution containing 2% PFA and 0.2% glutaraldehyde in PBS (pH 6.0) for 2 h on ice. Samples were washed three times with PBS and tissue was incubated overnight in SAβgal staining solution containing X-gal in N-N-dimethylformamide (pH 6.0) at 37 °C. The following day, the samples were washed with PBS then fixed in 4% PFA overnight at 4 °C. Tumor samples were subsenquently dehydrated with two consecutive steps in 50% and 70% ethanol and embedded in paraffin for serial sectioning. 5 μm sections were cut, counterstained with nuclear fast red (Sigma) or were processed for immunohistochemistry as described above with the exception that slides were counterstained for 30 s with nuclear fast red. Detection of SA-β-gal activity in cryosections was performed as previously described^63^.

### Western Blot analysis

Pituitary and GC tumor samples were lysed and homogenized in lysis solution (20mM Tris-HCl [pH 7.4], 150 mM NaCl, 1 mM EDTA, 1% Igepal, 0,1% SDS, 0,5% Deoxycholate, Complete Ultra protease inhibitors (Roche) and PhosphoSTOP phosphatase inhibitors (Roche). After SDS-PAGE separation, samples were transferred to PVDF membranes (BioRad). Immunoblots were developed using HRP-conjugated secondary antibodies (Jackson) and enhanced chemiluminescent (Thermo #34096). ImageQuant LAS 4000 Mini and Image Quant TL (GE Healthcare) were used for imaging and quantification. Antibodies used for immunoblotting were p16 (Santa Cruz Biotechnology #sc-1207) and p18 (Santa Cruz Biotechnology #sc-865). Anti-ß-actin (clone AC74, Sigma-Aldrich #A2228) was used as loading control.

### RNA isolation and Quantitative Real Time PCR

Nucleic acid isolation was performed using Absolutely RNA RT-PCR Miniprep Kit (Agilent, La Jolla, CA, USA) with deoxyribonuclease treatment, following manufacturer’s instruction. Total RNA purity and concentration was assessed using Nanodrop 2000 spectrophotometer (Thermo Scientific, Wilmington, NC, USA), and subsequently reverse-transcribed using random hexamer primers and the cDNA First Strand Synthesis kit (MRI Fermentas, Hanover, MD, USA). Quantitative PCR was performed using Brilliant III SYBR Green Master Mix in the Stratagene Mx3000p system (Agilent, La Jolla, CA, USA). Details regarding the development, validation and application of the qPCR methods in pituitary tissue have been previously described^64^,^65^. Briefly, in order to perform an absolute quantification of the starting copy number of cDNA for each sample examined using SYBR Green methods, reverse-transcribed samples derived from GC tumors and normal rat pituitary) were PCR amplified, and the signal was compared with that of an specific standard curve run in parallel on the same plate. Standard curves consisted of 1, 10^1^, 10^2^, 10^3^, 10^4^, 10^5^, and 10^6^ copies of synthetic cDNA template for each of the transcript examined. Standard curves were generated by the Stratagene Mx3000p Software, and the slope of a standard curve for each template examined was −3.31 and −3.35 (r^2^ of the standard curve between 0.997 and 0.999), indicating that the efficiency of amplification was close to 100%, meaning that all templates in each cycle were copied. qPCR thermal profile consisted of a first step at 95 °C/10 minutes, followed by 40 cycles of denaturation (95 °C/30 seconds), annealing (61 °C/60 seconds), and extension (72 °C/30 seconds); and finally, to verify that only one product was amplified, a dissociation cycle. To control for variations in the quantity of RNA used in the retro-transcription reaction and the efficiency of the RT-reaction, the expression level (expressed as copy-number) of each transcript of interest was adjusted by the expression of beta-actin [used as a housekeeping gene; mRNA levels of beta-actin did not significantly vary between experimental groups (data not shown)]. Specific primers sequence, GenBank accession numbers and product sizes for rat beta-actin and somatostatin receptors used in this study were as follows: beta-actin (Actb; forward 5′-CCTAAGGCCAACCGTGAAA-3′ and reverse 5′-CCAGAGGCATACAGGGACAA-3′, NM_031144.3, 104 bp), somatostatin receptor 1 (Sstr1; forward 5′-TGCCCTTTCTGGTCACTTCC-3′ and reverse 5′-AGCGGTCCACACTAAGCACA-3′, NM_012719.2, 135 bp), somatostatin receptor 2 (Sstr2; forward 5′-CCCATCCTGTACGCCTTCTT-3′ and reverse 5′-GTCTCATTCAGCCGGGATTT-3′, NM_019348.1, 134 bp), somatostatin receptor 3 (Sstr3; forward 5′- TTGGCCTCTACTTCCTGGTG-3′ and reverse 5′-ATCCTCCTCCTCCTCCGTCT-3′, NM_133522.1, 185 bp) and somatostatin receptor 5 (Sstr5; forward 5′-TCATTGTGGTCAAGGTGAAGG-3′ and reverse 5′-AAGAAATAGAGGCCGGCAGA-3′, NM_012882.2, 199 bp). Based on the stringent criteria to maximize specificity and efficiency, the use of qPCR can be used to accurately quantify copy number expression of mRNA transcripts, as previously reported^40^,^44^,^64^,^65^.

### Primary cell cultures and *in vitro* analysis

Primary tumor samples were dispersed into single cells by enzymatic/mechanical disruption, and cultured onto tissue culture plates in serum containing medium, as previously described^66^. Briefly, tumor samples were transferred to sterile culture medium (S-MEM, Gibco, Madrid, Spain) supplemented with 0.1% BSA, 0.01% L-glutamine, 1% antibiotic-antimycotic solution, and 2.5% HEPES. Tumors were minced into 1–2 mm^3^ pieces using sterile blades, washed and incubated in 30 mL S-MEM medium supplemented 0.3% trypsin (Beckson, Dickinson and Company, Sparks, MD, USA) for 2 h of gentle shaking at 37 °C in a spinner flask (Bellco Glass, Vineland, NJ, USA). Cells suspension was washed twice in 4.5 g/L glucose containing DMEM medium (Gibco, Madrid, Spain) complemented with 0.1% BSA, 0.01% L-glutamine, 1% antibiotic-antimycotic solution, and 2.5% HEPES. Cell viability (90–100% in all cases) was assessed by the Trypan blue exclusion test in a Neubauer chamber.

For *in vitro* cell proliferation experiments (10,000 cells/well were plated in a 96-well plates), SOM-230 (generously provided by Herbert A. Schmid; Novartis Pharma AG, Basel, Switzerland), octreotide (GP-Pharm, Barcelona, Spain) and sstr2 agonist (BIM-23120; generously provided by Michael D. Culler; IPSEN Bioscience, Cambridge, MA, USA) were administered at 100 nM, as previously reported^67^,^68^. Cell proliferation in response to the given treatments was evaluated using the MTT [3-(4,5-dimethylthiazol-2-yl)-2,5-diphenyltetrazolium bromide] assay. After 36 hours of culture, the medium was removed and fresh medium containing 1% FBS was added. After 24 h of incubation, treatments were added to the cells in 1% FBS containing medium. Proliferation rate was measured after 24, 48 and 72 hours of incubation. Before measuring, cells were pre-incubated for 2 hours with MTT-containing DPSS solution, medium was removed and DMSO solution with SDS and acetic acid was added to the cells and incubated in a shaker at 150 rpm for 15 minutes. Measurements were made at 590 nm using a Flex Station III (Molecular Devices, Madrid, Spain) coupled to a computer, and the software SoftMax Pro v5.4.1 (Molecular Devices, Madrid, Spain). DMEM containing 1% FBS was used as vehicle-control and positive control of inhibition of cell proliferation, respectively.

Similarly, for *in vitro* secretion experiments, 100,000 cells/well were plated in 24-well plates in FBS-containing medium. After 36 hours of culture, medium was replaced for FBS-free medium for 1 hour. Then, SOM-230, octreotide and sst2 agonist were administered at 100 nM for 4 h, using FBS-containing medium as a control, and media were collected and stored at −20 °C for further analysis by specific GH ELISA.

### Statistical analysis

For statistical comparisons two-tailed Student’s test or one-way and two-way ANOVA for repeated measures followed by post-hoc Tukey HSD and Bonferroni corrected t-tests. Data are presented as mean ± SEM. The Kaplan-Meier survival curves were used for survival analysis. Comparisons of survival curves were performed with the logrank test. A P-value less than 0.05 was considered significant.

## Additional Information

**How to cite this article**: Martín-Rodríguez, J. F. *et al.* Molecular Characterization of Growth Hormone-producing Tumors in the GC Rat Model of Acromegaly. *Sci. Rep.* **5**, 16298; doi: 10.1038/srep16298 (2015).

## Supplementary Material

Supplementary Information
