# Supplementary material for: Molecular Characterization of Growth Hormone-producing Tumors in the GC Rat Model of Acromegaly
Source: Sci Rep. 2015 Nov 9;5:16298. doi: 10.1038/srep16298 (PMC4637865; doi:10.1038/srep16298)
Supplement: Supplementary Information [file srep16298-s1.pdf]

Molecular Characterization of Growth Hormone-producing Tumors in the GC Rat Model of Acromegaly

Juan F. Martín-Rodríguez[1,2], Jose L. Muñoz-Bravo[1,2], Alejandro Ibañez-Costa[3], Laura Fernandez-Maza[4], Marcin Balcerzyk[4], Rocío Leal-Campanario[5], Raúl M. Luque[3], Justo P. Castaño[3], Eva Venegas-Moreno[1,2], Alfonso Soto-Moreno[1,2], Alfonso Leal-Cerro[2]*, David A. Cano[1,2]*

[1]Unidad de Gestión Clínica de Endocrinología y Nutrición, Hospital Universitario Virgen del Rocío, Sevilla. [2]Instituto de Biomedicina de Sevilla (IBiS), Hospital Universitario Virgen del Rocío/Consejo Superior de Investigaciones Científicas/Universidad de Sevilla, Sevilla (Spain).

[3]Departamento de Biología Celular, Fisiología e Inmunología. Universidad de Córdoba. Hospital Universitario Reina Sofía. Instituto Maimónides de Investigación Biomédica de Córdoba (IMIBIC). Campus de Excelencia Internacional Agroalimentario (ceiA3). CIBER Fisiopatología de la Obesidad y Nutrición (CIBERObn); Córdoba (Spain)

[4]Centro Nacional de Aceleradores (Universidad de Sevilla/CSIC/Junta de Andalucía), Sevilla, Spain

*These authors have codirected this study

Authors for correspondence: aleal@neuroendocrinologia.net, dcano-ibis@us.es

Supplementary Table 1. Splenomegaly in GC tumor-bearing rats compared to control rats.

Group	No.	Mean spleen weight (g)	Spleen-to-body weight ratio±SD	One-way ANOVA	Tukey HSD Post-hoc Test
Tumor-bearing group	12	1.79	0.006±0.002	$F_{(2,28)} = 14.22$ *p*<0.0001	Tumor-bearing vs. Tumorectorized group: *p*=0.0052 Tumor-bearing vs. Vehicle-treated group: *p*= 0.0001 Tumorectorized vs. Vehicle-treated group: *p*=0.5153
Tumorectorized group	7	0.78	0.003±0.002		
Vehicle-treated group	10	0.48	0.002±0.001		

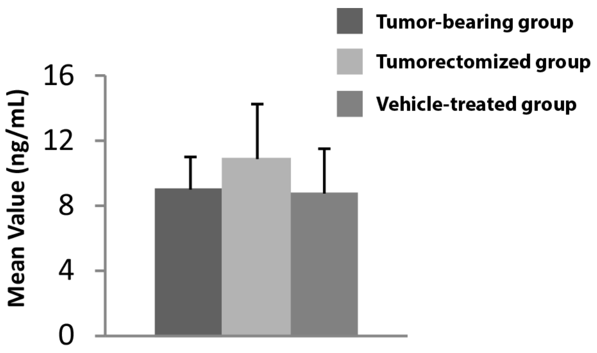

Supplementary Figure 1. Prolactin serum levels measured by Enzyme-linked immunosorbent assay (ELISA) data of prolactin in GC rats. No significant differences were found among the different groups.

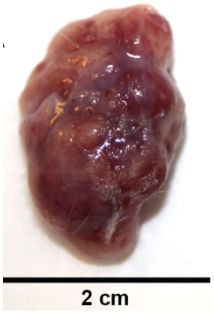

Supplementary Figure 2. Gross morphology of a GC tumor resected 5 weeks after subcutaneous injection of GC cells.

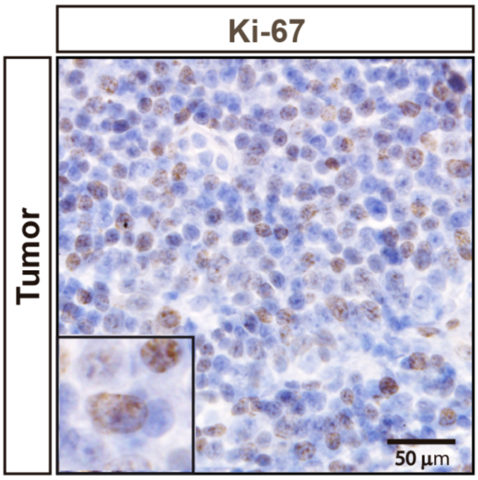

Subject	Field	Ki-67 + cells	Ki-67 - cells	Ki-67 LI ±SD
1	1	500	561	
	2	392	590	
	3	380	615	41.7 ±4.7
2	1	122	472	
	2	165	385	
	3	225	460	27.8 ±6.4
3	1	346	758	
	2	215	698	
	3	225	815	25.5 ±5.1
Total		2570	5354	**31.7 ±8.8**

Supplementary Figure 3. Ki-67 proliferative index in GC tumor. Top. Representative picture of a GC tumor section stained for the KI-67 antibody. Bottom. Ki-67 labelling index (LI) calculated in three independent GC tumors.

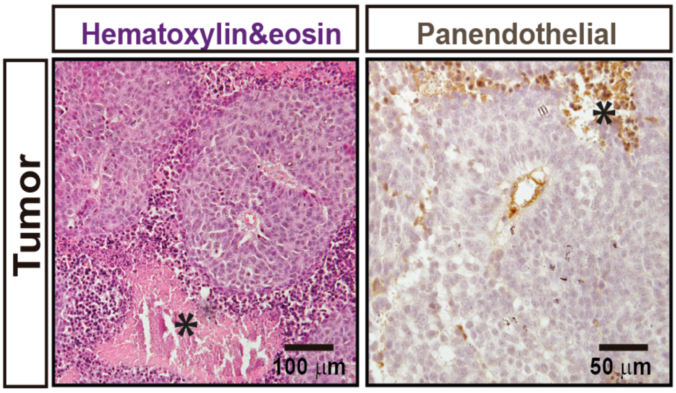

Supplementary Figure 4. Histological characterization of GC tumors at later stages. Left panel, Hematoxylin/eosin stained sections of GC tumors resected 10 weeks after subcutaneous injection of GC cells. Right panel, Immunohistochemical staining for the blood vessel marker PECAM-1 Asterisks denote necrotic areas.

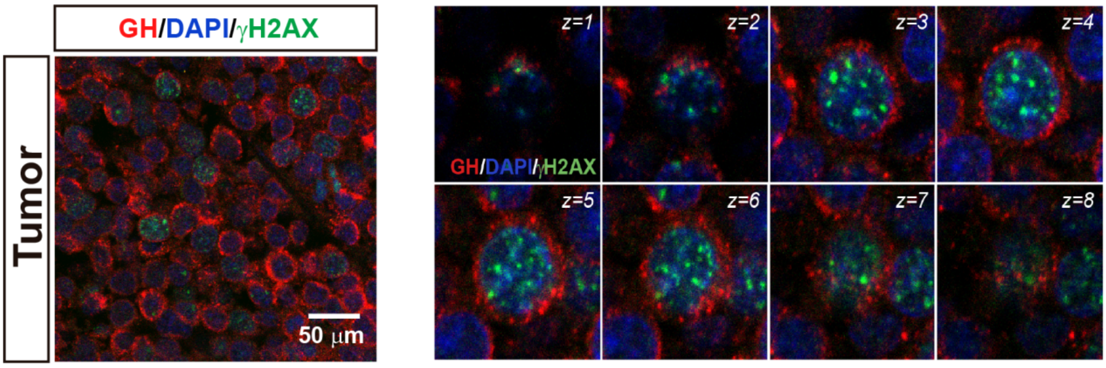

Supplementary Figure 5. Colocalization of γ-H2AX and growth hormone in GC tumor cells. Left panel, maximum intensity projection. Right panel, Z-stack confocal images through a GH-producing cell GC tumor depicting GH and γ-H2AX colocalization. Images were collected at 1 μm intervals using the 488, 561 and 405 lasers. These images was further used to create a 3D rendering using Imaris shown in panel H of Figure 4, DNA was counterstained with 4',6-diamidino-2-phenylindole (DAPI).

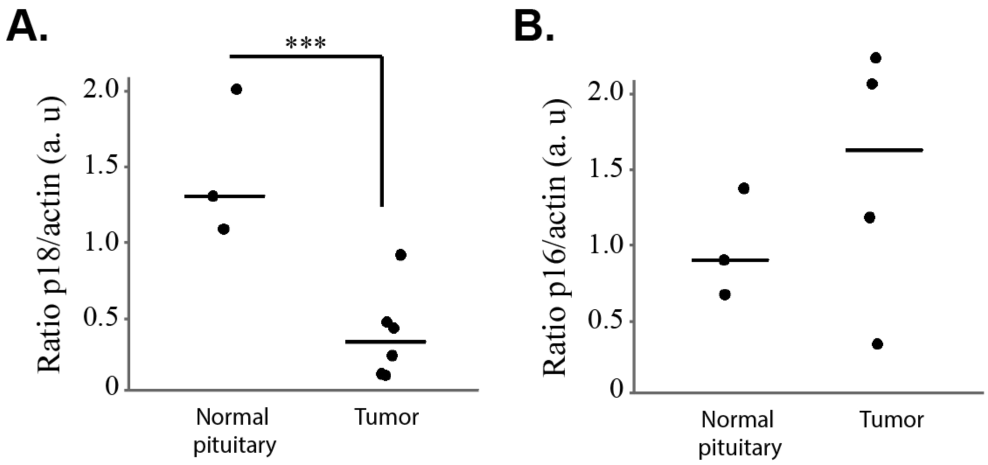

p

Supplementary Figure 6. Western blot quantification of (A) p18 and (B) p16 proteins in GC tumors and normal pituitary tissue. p18 levels were decreased in GC tumors while no significant differences were found in p16 protein levels between GC tumors and normal pituitary. ***$P < 0.001$ (Student's *t* test).

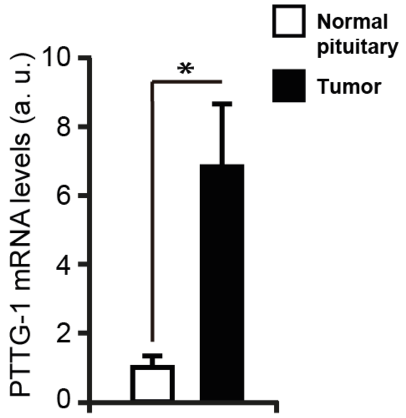

Supplementary Figure 7. Quantitative PCR analysis shows increased expression of PTTG1 in GC tumors compared to normal pituitary. n=5 samples per group. Data are mean ± s.e.m. *P<0.05.

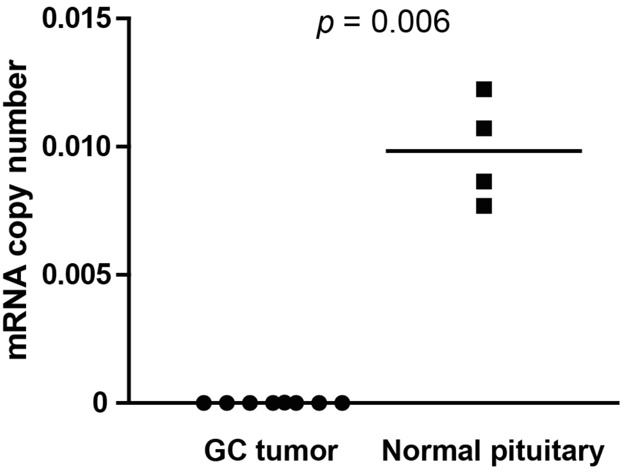

Supplementary Figure 8. Quantitative PCR analysis did not detect GHRH receptor expression in GC tumors. Solid lines denotes the group mean .

Antibody	Species	Dilution	Source (catalog number)
β-Catenin	Mouse	1:200	BD Transduction (610153)
γ-H2AX	Mouse	1:500	Merck Millipore (05-636)
ACTH	Rabbit	1:800	NHPP (AFP 156102789)
Caspase-3	Rabbit	1:200	Cell Signaling Technology (#9661)
E-Cadherin	Mouse	1:200	BD Transduction (610181)
GH	Rabbit	1:200	NHPP (AFP 5672099)
Ki67	Rabbit	1:200	Thermo Scientific (RM9106-S0)
Keratin 18	Mouse	1:200	Abcam (ab668)
Panendothelial cell antigen	Rat	1:300	BD Pharmigen (553849)
N-Cadherin	Rabbit	1:1000	Abcam (ab12221)
p21	Mouse	1:500	BD Pharmigen (556431)
p27	Mouse	1:400	BD Transduction (610241)
Prolactin	Rabbit	1:800	NHPP (AFP 107120402)
PTTG1	Mouse	1:50	Santa Cruz Biotechnology (sc-71947)

Supplementary Table 2. Primary antibodies
